# The American Association of Obstetricians and Gynecologists

**Published:** 1890-10

**Authors:** 


The American Association of Obstetricians and Gynecologists held its annual meeting at Philadelphia, Sept. 16th, 17th, and 18th. It was a largely attended and very successful meeting, and we expect to notice some of the work accomplished at a future time. The officers for the ensuing year are: President, Dr. A. H. Wright, Toronto; Vice-Presidents, Dr. George H, Rohé, Baltimore; Dr. R. B. Hall, Cincinnati; Secretary, Dr. Wm. Warren Potter, Buffalo; Treasurer, Dr. X. O. Werder, Pittsburg. Executive Council, Dr. Albert Vander Veer, Albany, Dr. Clinton Cushing, San Francisco, Dr. Charles A. L. Reed, Cincinnati, Dr. L. S. McMurtry, Louisville, Dr. R. L. Banta, Buffalo.

